# Intracellular proteins moonlighting as bacterial adhesion factors

**DOI:** 10.3934/microbiol.2018.2.362

**Published:** 2018-05-31

**Authors:** Constance Jeffery

**Affiliations:** Department of Biological Sciences, University of Illinois at Chicago, 900 S Ashland Ave, Chicago, IL 60607, USA

**Keywords:** moonlighting proteins, adhesion, multifunctional proteins

## Abstract

Pathogenic and commensal, or probiotic, bacteria employ adhesins on the cell surface to attach to and interact with the host. Dozens of the adhesins that play key roles in binding to host cells or extracellular matrix were originally identified as intracellular chaperones or enzymes in glycolysis or other central metabolic pathways. Proteins that have two very different functions, often in two different subcellular locations, are referred to as moonlighting proteins. The intracellular/surface moonlighting proteins do not contain signal sequences for secretion or known sequence motifs for binding to the cell surface, so in most cases is not known how these proteins are secreted or how they become attached to the cell surface. A secretion system in which a large portion of the pool of each protein remains inside the cell while some of the pool of the protein is partitioned to the cell surface has not been identified. This may involve a novel version of a known secretion system or it may involve a novel secretion system. Understanding the processes by which intracellular/cell surface moonlighting proteins are targeted to the cell surface could provide novel protein targets for the development of small molecules that block secretion and/or association with the cell surface and could serve as lead compounds for the development of novel antibacterial therapeutics.

## 1. Introduction to intracellular proteins that moonlight as bacterial adhesins

Bacterial adherence factors, also known as adhesins, are proteins on the cell surface that form and maintain physical interactions with host cells and tissues. They are important in both health and disease as they are needed by pathogens for infection and by commensal or “good” bacteria to maintain a symbiotic relationship with the host. Surprisingly, several dozen of these proteins were previously identified as ubiquitous intracellular enzymes that have a canonical function in essential cellular processes and are sometimes referred to as “housekeeping enzymes” [1]–[5]. The first intracellular/surface moonlighting protein (ISMP) to be identified was an enzyme in glycolysis, glyceraldehyde 3-phosphate dehydrogenase (GAPDH), which has a second role on the surface of pathogenic streptococci [6]. Other intracellular/surface moonlighting proteins include other metabolic enzymes that are also widespread in evolution and function in glycolysis, the citric acid cycle, or DNA and protein metabolism, for example, phosphoglycerate kinase and enolase. Intracellular chaperones (Hsp60/GroEL, Hsp70/DnaK), and protein synthesis elongation factors (EF-Tu, EF-G) have also been found to serve as adhesins in bacteria (Table 1).

In general, moonlighting proteins comprise a subset of multifunctional proteins that perform two or more distinct and physiologically relevant biochemical or biophysical functions that are not due to gene fusions, multiple RNA splice variants, or pleiotropic effects [1]. The MoonProt Database includes information about hundreds of moonlighting proteins for which biochemical or biophysical evidence supports the presence of at least two biochemical functions in one polypeptide chain [7]. Of these, over 30 types of proteins have one function inside the cell and another function as an adhesin on the cell surface. Some are found to moonlight on the surface of multiple species, so there are over 100 ISMPs. The bacterial ISMPs (Table 1) are found in typical Gram-positive and Gram-negative species, as well as mycobacteria, spirochetes, and mycoplasma.

**Figure 1.**
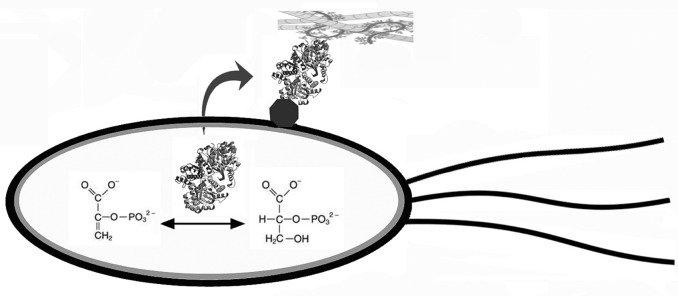
Intracellular enzymes and chaperones can function as adhesins on the bacterial cell surface. An ISMP can function as an enzyme inside of the cell and an adhesin when located on the cell surface. Enolase is found in the cytoplasm in almost all species where it converts 2-phosphoglycerate to phosphoenolpyruvate as the ninth step of glycolysis. In many species of bacteria, it is also found on the cell surface where it can bind to the host's extracellular matrix or airway mucins. For pathogenic bacteria, this attachment can be important for invading host tissues and promoting infection. In most cases, how the intracellular enzyme is transported outside the cell and how it becomes attached to the cell surface are not known (curved arrow). There might be a receptor for the protein on the bacterial cell surface (hexagon), but the nature of the surface attachment is known for only a few ISMPs.

An ISMP can have different extracellular functions in different species. Enolase converts the reversible conversion of 2-phosphoglycerate to phosphoenolpyruvate in the cytoplasm in glycolysis and gluconeogenesis and has been found to have many moonlighting functions in addition to being an adhesin on the bacterial cell surface. As a bacterial adhesin, enolase binds host proteins in the extracellular matrix, mucin, and other proteins and plays an important role in infection of mammalian and avian hosts [8]–[24] (Figure 1). Some ISMPs also have third (or more) functions as secreted soluble proteins, in many cases with roles in modulation of the immune system [2],[3].

## 2. Importance in health and disease

In pathogenic bacteria the extracellular function often plays a key role in infection or virulence [2],[3]. ISMPs have been found to be involved in aiding the bacteria to bind directly to host cells, including fructose-1,6-bisphosphate aldolase from *Neisseria meningitidis* [25] and *Streptococcus pneumoniae* [26] and the Hsp60 chaperone from *Clostridium difficile* [27], *Helicobacter pylori* [28], *Chlamydia pneumoniae* [29], *Legionella pneumophila* [30] and several other species. In some cases a specific receptor on the host cell surface has been identified. *Listeria monocytogenes* alcohol acetaldehyde dehydrogenase binds to Hsp60 (another moonlighting protein) on the surface of several human cell lines [31],[32]. *Streptococcus pyogenes* GAPDH binds to the uPAR/CD87 receptor [33]. *Streptococcus pneumoniae* fructose 1,6-bisphosphate aldolase binds to the flamingo cadherin receptor (FCR) [26]. *Haemophilus ducreyi* Hsp60 binds to membrane glycosphingolipids [34],[35].

Other ISMPs bind to extracellular matrix or secreted mucins in the mucosal layer of the intestines and airway. *Mycoplasma pneumoniae* EF-Tu and pyruvate dehydrogenase, *Mycobacterium tuberculosis* malate synthase, and *Streptococcus mutans* autolysin AltA, *Staphylococcus caprae* autolysin AltC, and *Staphylococcus aureus* autolysin Aaa bind to one or more of the extracellular matrix components fibronectin, laminin, and/or collagen [36]–[40]. *Mycoplasma genitalium* GAPDH, *Salmonella typhimurium* Hsp60, and *Streptococcus gordonii* enolase, EF-Tu, and the beta subunit of the DNA-directed RNA polymerase bind mucin [18],[41],[42]. Other examples are given in Table 1.

The ability of many pathogenic bacteria to use surface proteins to bind to the soluble host protein plasminogen also assists in invasion of host tissues [11]–[14],[43],[44]. Plasminogen is a precursor to plasmin, which is a broad-spectrum serine protease present in blood that helps break down fibrin clots [45]. When an invading pathogen uses a receptor on its surface to bind plasminogen from the host, the plasminogen can be converted to plasmin, the active form of the protease, by using an endogenous protease or subverting the host's tissue-type plasminogen (tPA) activators and urokinase-type plasminogen activators [46]. The active plasmin that is then attached to the surface of the invading organism can be used as a general protease to degrade host extracellular matrix and basement membrane, thereby facilitating migration through tissues. In the case of *Mycoplasma hyopenumoniae*, a swine-specific pathogen with a reduced genome that lacks genes for building amino acids, having an active protease on the surface enables cleavage of a variety of host proteins to produce peptides and amino acids that can be taken up by the bacterium as nutrients [47],[48]. Other ISMPs also aid in infection and virulence by serving as receptors on the bacterial cell surface to acquire nutrients from the host. Staphylococcal GAPDH serves as a transferrin binding protein to acquire iron from the host [49].

The use of moonlighting proteins in adherence to host cells and tissues is not seen only in pathogenic species. Bacterial species that are sometimes referred to as “good” bacteria or probiotics, in other words nonpathogenic symbionts that help promote health and well-being, use ISMP in commensal interactions with host species, especially in the intestines. *Lactobacillus plantarum* GAPDH and enolase were shown to aid the bacterium in binding to mammalian cells and could play a role for this probiotic species to bind to the lining of the gut [23],[50]. *Lactobacillus johnsonii* EF-Tu and Hsp60 also bind to human cells and to mucin [51],[52]. *Lactobacillus acidophilus* GAPDH also binds mucin [53].

ISMPs may also assist in symbiotic relationships with other species, including a symbiotic relationship between lactic acid bacteria and yeast. The bacteria break down starch and other carbohydrates to produce lactic acid that is used by the yeast. In return they receive nutrients made by the yeast. This symbiotic relationship is found in several kinds of fermented foods like kefir, a drink made from cow's milk. *Lactococcus lactis* GAPDH, pyruvate kinase, Hsp60/GroEL, DnaK/Hsp70, and 6-phosphofructokinase have been shown to bind to invertase on the surface of the yeast *Saccharomyces cerevisiae* to help maintain this inter-species symbiotic interaction [54].

One of the benefits of probiotic bacteria has been suggested to be that they compete with pathogens for binding sites or nonspecific binding to the surface of epithelial cells lining the gut. Several moonlighting proteins were found to aid Lactobacillus species in competing with pathogenic species for binding to epithelial cell lines in vitro. Some of the same ISMPs may be involved in the competition of pathogenic and commensal bacteria for binding to epithelial cells. Several of the moonlighting proteins have been found to perform the same combination of enzyme and adhesion functions in both pathogenic and commensal bacteria, for example, enolases from Lactobacillus, Staphylococcus, Streptococcus and several other species bind human plasminogen [14].

## 3. Proteomic and other technical approaches for identifying intracellular/surface proteins

The adhesion functions of the ISMPs in Table 1 were mainly found through experiments to identify proteins that bind to a specific molecular target, such as collagen, fibronectin, or other extracellular matrix proteins or through studies of proteins involved in binding to a specific target cell type. In recent years, many more intracellular proteins have been found to have a second location on the cell surface through surface proteomics, or “surfomics”, studies that aimed to identify all the proteins on a cell surface [55]. Surface proteomics studies employ variations of three types of experimental approaches to identify cell surface proteins. The main difference in the methods is in how the candidate proteins are isolated: through fractionating the cells to isolate components of the cell membrane and/or cell wall, surface “shaving” or using proteases to digest proteins on the cell surface without damaging the cell membrane, or labeling proteins on the surface with biotin or O^18^ before disrupting the cells and isolating the proteins. In each case the surface proteins are then identified using mass spectrometry. Although these methods might incorrectly identify some strictly intracellular proteins as being part of the cell surface proteome due to experimental artifacts inherent in the challenges of cell fractionation, and even some intracellular proteins that are correctly found to have a second location on the cell surface might have a different function other than as adhesins, at least some of the known intracellular/surface adhesins were correctly found to be localized to the cell surface, and it is possible that some of the additional cytoplasmic proteins found in these studies are also moonlighting as adhesins. Additional experiments are needed to determine if the intracellular proteins identified as being on the cell surface through proteomics methods are indeed involved in bacterial adhesion and were not found on the surface because they have another role on the surface or perhaps they were artifacts of the experimental methods.

## 4. Molecular mechanisms for intracellular proteins to function as cell surface adhesins

It might at first seem unlikely that so many intracellular chaperones and enzymes required for central metabolism evolved to function also as cell surface binding proteins. Acquiring the new function required (1) evolution of a new protein-protein interaction site as well as (2) mechanisms for secretion and cell surface attachment, all while maintaining the first function of the protein. Satisfying the first requirement can be surprisingly simple. In general, most of the amino acid residues on a protein's surface are not directly connected to the protein's main function and are therefore not under significant selective pressure during evolution. In fact, surface amino acids vary a great deal even among close homologues. Having just a small number of these surface residues in a correct three-dimensional arrangement can be sufficient for formation of a novel protein-protein interaction site. In fact, Ehinger and coworkers showed that a nine amino acid sequence on the surface of enolase was sufficient for its interaction with plasminogen [56]. In general, for an average protein comprised of 300 or 400 amino acids, there is ample space and material for development of a new protein-protein binding site. In addition, most of these proteins are essential housekeeping proteins that first evolved billions of years ago and are expressed in many species and cell types, providing both the time and variety of cellular conditions for evolution of the protein surface to include a new binding function.

A more difficult question is how most of the ISMPs are secreted and become attached to the cell surface. The ISMPs do not contain a signal sequence or the twin arginine motif found in most proteins secreted by the canonical Sec or TAT secretion systems, respectively. For these reasons, the ISMPs are sometimes referred to as anchorless surface proteins or surface-associated housekeeping enzymes and are said to be secreted through non-classical, noncanonical, or unconventional secretion pathways. It is not clear if any of the known non-canonical secretion systems are involved in the secretion of ICMS, but most still require a kind of secretion signal, and they tend to be involved in the secretion of a few specific proteins [57].

Although it has been suggested that these intracellular proteins could become released from dead or damaged cells, several lines of evidence support the idea that at least some of them do require a secretion system [58],[59]. First, the ISMPs are not the most abundant proteins in the cell, and those proteins that are most abundant are not often found on the cell surface. Second, a large portion of the pool of each protein type remains inside the cell while only some of the pool of the protein is partitioned to the cell surface. Why only part of the cytoplasmic pool of these specific proteins become targeted to the cell surface is not known.

For some individual proteins, there is additional evidence that a secretion system is probably involved. Yang and coworkers concluded that the release of GroEL, DnaK, enolase, pyruvate dehydrogenase subunits PdhB and PdhD, and superoxide dismutase SodA, by *Bacillus subtilis* is not due to gross cell lysis based on observing a constant cell density, no change in secretion in the presence of chloramphenicol, constant cell viability count, negligible amounts of two highly expressed cytoplasmic proteins EF-Tu and SecA in the culture medium, and the lack of effect of deleting lytC and lytD autolysins on the amount of the proteins in the media [60]. They also showed that these proteins were not released into the medium by membrane vesicles and there was no N-terminal cleavage (which might have suggested the presence of a signal sequence). Also, a mutant form of enolase with a hydrophobic helix replaced with a more neutral helix was retained in the cell when the wild type protein was found in the media, which also supports the model that it is not due to cell lysis. They followed up by showing that in *Bacillus subtilis* enolase, the internal hydrophobic helical domain was essential but not sufficient for export of enolase [61], although a larger portion of the N-terminal domain (residues 1–140) was sufficient for export of GFP in *B subtilis* and *E coli.* Boel and colleagues found that Lys341 of *E. coli*, *Enterococcus faecalis*, and *Bacillus subtilis* enolase becomes spontaneously modified with the substrate 2-phosphoglycerate (2PG), and this post-translational modification is required for export form the cell [62]. Substitution of Lys341 with other amino acids (Ala, Arg, Glu, Gln) prevented modification and secretion even though the Lys341Glu mutant enzyme was enzymatically active, showing that enzyme activity was not sufficient for secretion (and also that secretion was not due to cell leakage, because a single amino acid change can cause a decrease in secretion).

Secretion of some ISMPs may involve an as yet unknown secretion pathway, or their secretion might utilize an alternative version of one or more of the known secretion systems. If the latter is true, there are several possibilities, which are not mutually exclusive: One or more of the known secretion systems could be leaky. Post translational modifications (PTMs), possibly transient PTMs, can render some subset of the ISMP to be passable substrates. Alternative versions of the secretion systems might exist that require additional proteins such as a chaperone that have not yet been identified. An alternative system's secretion could be in competition with folding/unfolding or only rare conformations of an ISMP might be competent for secretion. It's possible that some combination of these factors could result in an inefficient secretion process, or that the alternative version requires induction of the expression of an unknown protein component of a known secretion system or an enzyme involved in adding PTMs. A search for shared characteristics might suggest what protein features singled out these intracellular proteins for adoption to play a second role on the cell surface, but a study of 98 ISMPs found that they share physical characteristics typical of intracellular proteins [63]. A couple studies have identified peptides on the cell surface that are the results of proteolytic cleavage of intracellular proteins, including EF-Tu [64],[65], and the authors suggested that cleavage might yield peptides that are better at binding to some host proteins than the intact ISMPs. Because intact versions of these proteins are also found on the cell surface, the proteolytic cleavage is likely to take place after transport across the membrane and not as part of the secretion mechanism.

After the intracellular proteins are transported to the extracellular milieu, they become anchored to the surface of the bacterial cells, but in most cases, the mechanism for cell surface anchoring is also not known. For surface proteins in general, known anchoring mechanisms involve an N-terminal signal sequence for secretion and/or a C-terminal sorting motif, such as the LPXTG motif that is recognized by sortase A, for anchoring to the peptidoglycan network on the cell surface [66]. A smaller number of surface proteins have been found to be targeted to the cell surface due to the presence of additional motifs [67]–[69], including the GW repeat, the choline binding motif, and the LysM domain, but these are not found in the majority of the ISMPs in Table 1. Studies with purified proteins have shown that some intracellular/surface moonlighting proteins can adhere to the cell surface by re-association in both Gram-positive and Gram-negative bacteria, so it is possible that some of the ISMPs are secreted and then re-associate with the cell surface of after secretion. An increase in extracellular pH has been shown to cause some *Lactobacillus crispatus* ISMPs to be released from the cell surface [70]. Some ISMPs may also be released from the surface during cell-wall renewal that occurs during exponential growth phase [71]. In most cases it is not known to which components of the cell surface—proteins, lipids, etc.—the proteins bind, but it was shown recently that extracellular enolase is bound to a rhamnose residue in cell membrane of mycoplasma [72], and enolase and GAPDH bind covalently to lipotectoic acid on *Lactobacillus* *crispatus* [73].

## 5. Potential for targeting ISMPs in the development of novel antibacterials and treatments for IBD

With the increasing problem of antibiotic resistance [74],[75], new methods for inhibiting bacterial infections and virulence are needed, and studies of ISMPs might provide new targets for the development of novel therapeutics. But it's not the moonlighting proteins themselves that might be the best targets. The catalytic mechanisms of most of these ISMP are conserved between bacteria and their human hosts, which makes sense because they play key roles in central metabolic pathways such as glycolysis. Instead of targeting the ISMPs, elucidating how these proteins are targeted to the bacterial cell surface might identify processes and proteins that are involved in the novel secretion systems (or new versions of known secretion systems) or surface attachment mechanisms and that could serve as novel targets for developing new strategies for controlling infection.

Learning how pathogenic and commensal bacteria adhere to host cells and tissues could also lead to better understanding of how these species colonize host tissues and compete with each other. This information can be important in treatment of diseases that involve an imbalance of pathogenic and probiotic bacterial species, for example ulcerative colitis and Crohn's disease [76], which are autoimmune diseases of the gut that affect over a million people in the US alone [77]. Understanding bacterial adhesion could potentially lead to information about how probiotic species could be used to displace pathogens and improve the balance of bacterial species.

**Table 1.** Intracellular proteins that function as cell surface adhesins in bacteria.

Protein	Species	UniProt ID	Extracellular function	References
6-phosphofructokinase	*Lactococcus lactis*	P0DOB5	yeast invertase	[54]
	*Streptococcus oralis*	E6KMA1	plasminogen	[78]
Aaa autolysin	*Staphylococcus aureus*	Q2YVT4	fibronectin	[37]
Aae autolysin	*Staphylococcus epidermis*	Q8CPQ1	fibrinogen, fibronectin, vitronectin	[79]
Aspartase	*Haemophilus influenzae*	P44324	plasminogen	[80]
Atla autolysin	*Streptococcus mutans*	U3SW74	fibronectin	[39]
AtlC autolysin	*Staphylococcus caprae*	Q9AIS0	fibronectin	[40]
Bile salt hydrolase	*Bifidobacterium lactis*	Q9KK62	plasminogen	[81]
C5a peptidase	*Streptococcus agalactiae*	Q8E4T9	fibronectin	[82]
DNA-directed RNA polymerase beta subunit	*Streptococcus gordonii*	A0EKJ1	Muc7	[18]
DnaK	*Bifidobacterium*	Q8G6W1	plasminogen	[81]
	*Lactococcus lactis*	P0A3J0	yeast invertase	[54]
	*Mycobacterium tuberculosis*	A0A0H3L5C8	plasminogen	[75]
	*Neisseria meningitidis*	A9M296	plasminogen	[20]
EF-Tu	*Lactobacillus johnsonii*	Q74JU6	cells, mucins	[51]
	*Mycoplasma pneumoniae*	P23568	fibronectin, epithelial cells, plasminogen, heparin, fetuin, actin, fibrinogen, vitronectin, laminin	[36],[64]
	*Pseudonomas aeruginosa*	P09591	plasminogen	[43]
	*Streptococcus gordonii*	A8AWA0	Muc7	[18]
Elongation factor G	*Streptococcus gordonii*	A8AUR6	Muc7	[18]
Endopeptidase O	*Streptococcus pneumoniae*	Q8DNW9	plasminogen, fibronectin	[83]
Enolase	*Aeromonas hydrophila*	Q8GE63	plasminogen	[22]
	*Bacillus anthracis*	D8H2L1	plasminogen, laminin	[8]
	*Bifidobacterium lactis*	B7GTK2	plasminogen	[11]
	*Borrelia burgdorferi*	B7J1R2	plasminogen	[16]
	*Lactobacillus crispatus*	Q5K117	plasminogen, laminin	[14]
	*Lactobacillus johnsonii*	Q74K78	plasminogen, laminin	[14]
	*Lactobacillus plantarum*	Q88YH3	fibronectin	[23]
	*Leishmania mexicana*	Q3HL75	plasminogen	[13]
	*Mycoplasma fermentans*	C4XEI3	plasminogen	[24]
	*Mycoplasma suis*	F0QRW4	red blood cells	[84]
	*Mycoplasma synoviae*	Q4A740	plasminogen, fibronectin	[9]
	*Neisseria meningitidis*	E0N8L2	plasminogen	[20]
	*Staphylococcus aureus*	Q6GB54	plasminogen, laminin	[14],[21]
	*Streptococcus canis*	I7WI49	plasminogen	[17]
	*Streptococcus gordonii*	A8AY46	Muc7	[18]
	*Streptococcus mutans*	Q8DTS9	plasminogen	[12]
	*Streptococcus oralis*	A0A1F1EC06	plasminogen	[19]
	*Streptococcus pneumoniae*	Q97QS2	plasminogen	[14]
	*Streptococcus pyogenes*	Q1JML5	plasminogen	[14]
	*Streptococcus suis*	A4W2T1	fibronectin, plasminogen	[15]
Fructose 1,6-bisphosphate aldolase	*Neisseria meningitidis*	F0N9L0	cells	[25]
GAPDH	*Bacillus anthracis*	Q81X74	plasminogen	[85]
	*Lactobacillus acidophilus*	Q5FL51	mucin	[53]
	*Lactobacillus plantarum*	F9UM10	mucin, Caco-2 cells	[50]
	*Lactococcus lactis*	P52987	yeast invertase	[54]
	*Mycoplasma genitalium*	P47543	mucin	[41]
	*Staphylococcus aureus*	Q6GIL8	transferrin	[49]
	*Streptococcus agalactiae*	Q9ALW2	plasminogen	[86]
	*Streptococcus oralis*	A0A0F2E7M6	plasminogen	[78]
	*Streptococcus pneumoniae*	A0A0H2US80	plasminogen	[87]
	*Streptococcus pyogenes*	P68777	uPAR/CD87 receptor on human cells, plasminogen	[33],[88]
	*Streptococcus suis*	Q3Y454	plasminogen	[89]
Glucose 6-phosphate isomerase	*Lactobacillus crispatus*	K1MKZ7	laminin, collagen	[90]
Glutamine synthetase	*Lactobacillus crispatus*	D5GYN9	fibronectin, laminin, collagen I, plasminogen	[90]
	*Mycobacterium tuberculosis*	A0A0H3LHU4	plasminogen, fibronectin	[91]
	*Bifidobacterium lactis*	C2GUH0	plasminogen	[81]
Hsp60	*Chlamydiae pneumoniae*	P31681	adhesin	[29]
	*Lactococcus lactis*	P37282	yeast invertase	[54]
	*Legionella pneumophila*	Q5X762	adhesin	[30]
	*Clostridium difficile*	Q9KKF0	adhesin	[27]
	*Haemophilus ducreyi*	P31294	glycosphinngolipids	[34],[35]
	*Helicobacter pylori*	Q8RNU2	adhesin	[28]
	*Lactobacillus johnsonii*	F7SCR2	adhesin	[52]
	*Listeria*	Q8KP52	adhesin	[32]
	*Salmonella typhimurium*	P0A1D3	mucus	[42]
Hsp65/Cpn60.2/GroEL2	*Mycobacterium tuberculosis*	A0A0H3LCC3	CD43 on macrophage surface	[92]
Leucyl aminopeptidase	*Mycoplasma hyopneumoniae*	Q4A9M4	heparin	[93]
Malate synthase	*Mycobacterium tuberculosis*	P9WK17	fibronectin, laminin, epithelial cells	[38]
Glutamyl aminopeptidase	*Mycoplasma hyopneumoniae*	Q4AAK4	plasminogen, heparin	[47]
Leucyl aminopeptidase	*Mycoplasma hyopneumoniae*	Q4A9M4	plasminogen, heparin, DNA	[48]
Ornithine carbamoyltransferase	*Staphylococcus epidermidis*	P0C0N1	fibronectin	[94]
Peroxiredoxin	*Neisseria meningitidis*	A0A125WDU3	plasminogen	[20]
	*Streptococcus agalactiae*	E7S2A7	heme	[95]
Phosphoglycerate kinase	*Streptococcus oralis*	A0A0G7HBY7	plasminogen	[77]
	*Streptococcus agalactiae*	Q8DXT0	plasminogen, actin	[83],[96]
	*Streptococcus pneumoniae*	Q8DQX8	plasminogen	[97]
Phosphoglycerate mutase	*Bifidobacterium lactis*	P59159	plasminogen	[81]
	*Streptococcus oralis*	E6IYJ0	plasminogen	[78]
Pyruvate dehydrogenase	*Mycoplasma pneumoniae*	P75391	fibrinogen	[36]
Pyruvate kinase	*Lactococcus lactis*	Q07637	yeast invertase	[54]
Superoxide dismutase	*Mycobacterium avium*	P53647	adhesin	[98]
Triose phosphate isomerase	*Streptococcus oralis*	E6J203	plasminogen	[78]

## 6. Conclusions

The large number of ISMPs, the variety of bacterial species, and the different host proteins targeted suggests that this phenomenon of intracellular housekeeping proteins moonlighting as adhesins on the bacterial cell surface is widespread. There is still a great deal to learn about these proteins, especially how these intracellular proteins are secreted and attached to the bacterial cell surface. Studies of ISMP that serve as adhesins could help in identifying novel targets for development of therapeutics because their mechanisms of secretion and membrane attachment are likely to involve new proteins and cellular processes.

## References

[1] Jeffery CJ (1999). Moonlighting proteins. Trends Biochem Sci.

[2] Henderson B, Martin A (2011). Bacterial virulence in the moonlight: multitasking bacterial moonlighting proteins are virulence determinants in infectious disease. Infect Immun.

[3] Henderson B, Martin A (2013). Bacterial moonlighting proteins and bacterial virulence. Curr Top Microbiol.

[4] Jeffery CJ (2009). Moonlighting proteins—an update. Mol Biosyst.

[5] Kainulainen V, Korhonen TK (2014). Dancing to another tune-adhesive moonlighting proteins in bacteria. Biology.

[6] Pancholi V, Fischetti VA (1992). A major surface protein on group A streptococci is a glyceraldehyde-3-phosphate-dehydrogenase with multiple binding activity. J Exp Med.

[7] Mani M, Chen C, Amblee V (2015). MoonProt: a database for proteins that are known to moonlight. Nucleic Acids Res.

[8] Agarwal S, Kulshreshtha P, Bambah MD (2008). Alpha-enolase binds to human plasminogen on the surface of *Bacillus anthracis*. BBA-Proteins Proteom.

[9] Bao S, Guo X, Yu S (2014). *Mycoplasma synoviae* enolase is a plasminogen/fibronectin binding protein. BMC Vet Res.

[10] Boleij A, Laarakkers CM, Gloerich J (2011). Surface-affinity profiling to identify host-pathogen interactions. Infect Immun.

[11] Candela M, Biagi E, Centanni M (2009). Bifidobacterial enolase, a cell surface receptor for human plasminogen involved in the interaction with the host. Microbiology.

[12] Jones MN, Holt RG (2007). Cloning and characterization of an alpha-enolase of the oral pathogen *Streptococcus mutans* that binds human plasminogen. Biochem Bioph Res Co.

[13] Vanegas G, Quiñones W, Carrasco-López C (2007). Enolase as a plasminogen binding protein in *Leishmania mexicana*. Parasitol Res.

[14] Antikainen J, Kuparinen V, Lähteenmäki K (2007). Enolases from Gram-positive bacterial pathogens and commensal lactobacilli share functional similarity in virulence-associated traits. FEMS Immunol Med Mic.

[15] Esgleas M, Li Y, Hancock MA (2008). Isolation and characterization of alpha-enolase, a novel fibronectin-binding protein from *Streptococcus suis*. Microbiology.

[16] Floden AM, Watt JA, Brissette CA (2011). *Borrelia burgdorferi* enolase is a surface-exposed plasminogen binding protein. PLoS One.

[17] Fulde M, Rohde M, Polok A (2013). Cooperative plasminogen recruitment to the surface of *Streptococcus canis* via M protein and enolase enhances bacterial survival. MBio.

[18] Kesimer M, Kilic N, Mehrotra R (2009). Identification of salivary mucin MUC7 binding proteins from *Streptococcus gordonii*. BMC Microbiol.

[19] Kinnby B, Booth NA, Svensater G (2008). Plasminogen binding by oral streptococci from dental plaque and inflammatory lesions. Microbiology.

[20] Knaust A, Weber MV, Hammerschmidt S (2007). Cytosolic proteins contribute to surface plasminogen recruitment of *Neisseria meningitidis*. J Bacteriol.

[21] Carneiro CR, Postol E, Nomizo R (2004). Identification of enolase as a laminin-binding protein on the surface of *Staphylococcus aureus*. Microbes Infect.

[22] Sha J, Erova TE, Alyea RA (2009). Surface-expressed enolase contributes to the pathogenesis of clinical isolate SSU of *Aeromonas hydrophila*. J Bacteriol.

[23] Castaldo C, Vastano V, Siciliano RA (2009). Surface displaced alfa-enolase of *Lactobacillus plantarum* is a fibronectin binding protein. Microb Cell Fact.

[24] Yavlovich A, Rechnitzer H, Rottem S (2007). Alpha-enolase resides on the cell surface of *Mycoplasma fermentans* and binds plasminogen. Infect Immun.

[25] Tunio SA, Oldfield NJ, Berry A (2010). The moonlighting protein fructose-1, 6-bisphosphate aldolase of *Neisseria meningitidis*: surface localization and role in host cell adhesion. Mol Microbiol.

[26] Blau K, Portnoi M, Shagan M (2007). Flamingo cadherin: a putative host receptor for *Streptococcus pneumoniae*. J Infect Dis.

[27] Hennequin C, Porcheray F, Waligora-Dupriet A (2001). GroEL (Hsp60) of *Clostridium difficile* is involved in cell adherence. Microbiology.

[28] Yamaguchi H, Osaki T, Kurihara N (1997). Heat-shock protein 60 homologue of *Helicobacter pylori* is associated with adhesion of *H. pylori* to human gastric epithelial cells. J Med Microbiol.

[29] Wuppermann FN, Molleken K, Julien M (2008). Chlamydia pneumoniae GroEL1 protein is cell surface associated and required for infection of HEp-2 cells. J Bacteriol.

[30] Garduno RA, Garduno E, Hoffman PS (1998). Surface-associated hsp60 chaperonin of *Legionella pneumophila* mediates invasion in a HeLa cell model. Infect Immun.

[31] Jagadeesan B, Koo OK, Kim KP (2010). LAP, an alcohol acetaldehyde dehydrogenase enzyme in Listeria, promotes bacterial adhesion to enterocyte-like Caco-2 cells only in pathogenic species. Microbiology.

[32] Wampler JL, Kim KP, Jaradat Z (2004). Heat shock protein 60 acts as a receptor for the Listeria adhesion protein in Caco-2 cells. Infect Immun.

[33] Jin H, Song YP, Boel G (2005). Group A streptococcal surface GAPDH, SDH, recognizes uPAR/CD87 as its receptor on the human pharyngeal cell and mediates bacterial adherence to host cells. J Mol Biol.

[34] Frisk AC, Ison CA, Lagergard T (1998). GroEL heat shock protein of *Haemophilus ducreyi*: association with cell surface and capacity to bind to eukaryotic cells. Infect Immun.

[35] Pantzar M, Teneberg S, Lagergard T (2006). Binding of *Haemophilus ducreyi* to carbohydrate receptors is mediated by the 58.5-kDa GroEL heat shock protein. Microbes Infect.

[36] Dallo SF, Kannan TR, Blaylock MW (2002). Elongation factor Tu and E1 beta subunit of pyruvate dehydrogenase complex act as fibronectin binding proteins in *Mycoplasma pneumoniae*. Mol Microbiol.

[37] Heilmann C, Hartleib J, Hussain MS (2005). The multifunctional *Staphylococcus aureus* autolysin aaa mediates adherence to immobilized fibrinogen and fibronectin. Infect Immun.

[38] Kinhikar AG, Vargas D, Li H (2006). *Mycobacterium tuberculosis* malate synthase is a laminin-binding adhesin. Mol Microbiol.

[39] Jung CJ, Zheng QH, Shieh YH (2009). *Streptococcus mutans* autolysin AtlA is a fibronectin-binding protein and contributes to bacterial survival in the bloodstream and virulence for infective endocarditis. Mol Microbiol.

[40] Allignet J, England P, Old I (2002). Several regions of the repeat domain of the *Staphylococcus caprae* autolysin, AtlC, are involved in fibronectin binding. FEMS Microbiol Lett.

[41] Alvarez RA, Blaylock MW, Baseman JB (2003). Surface localized glyceraldehyde-3-phosphate dehydrogenase of *Mycoplasma genitalium* binds mucin. Mol Microbiol.

[42] Ensgraber M, Loos M (1992). A 66-kilodalton heat shock protein of *Salmonella typhimurium* is responsible for binding of the bacterium to intestinal mucus. Infect Immun.

[43] Kunert A, Losse J, Gruszin C (2007). Immune evasion of the human pathogen *Pseudomonas aeruginosa*: elongation factor Tuf is a factor H and plasminogen binding protein. J Immunol.

[44] Raymond BB, Djordjevic S (2015). Exploitation of plasmin(ogen) by bacterial pathogens of veterinary significance. Vet Microbiol.

[45] Collen D, Verstraete M (1975). Molecular biology of human plasminogen II Metabolism in physiological and some pathological conditions in man. Thromb Diath Haemorrh.

[46] Dano K, Andreasen PA, Grondahl-Hansen J (1985). Plasminogen activators, tissue degradation, and cancer. Adv Cancer Res.

[47] Robinson MW, Buchtmann KA, Jenkins C (2013). MHJ_0125 is an M42 glutamyl aminopeptidase that moonlights as a multifunctional adhesin on the surface of *Mycoplasma hyopneumoniae*. Open Biol.

[48] Jarocki VM, Santos J, Tacchi JL (2015). MHJ_0461 is a multifunctional leucine aminopeptidase on the surface of *Mycoplasma hyopneumoniae*. Open Biol.

[49] Modun B, Williams P (1999). The staphylococcal transferrin-binding protein is a cell wall glyceraldehyde-3-phosphate dehydrogenase. Infect Immun.

[50] Kinoshita H, Uchida H, Kawai Y (2008). Cell surface *Lactobacillus plantarum* LA 318 glyceraldehyde-3-phosphate dehydrogenase (GAPDH) adheres to human colonic mucin. J Appl Microbiol.

[51] Granato D, Bergonzelli GE, Pridmore RD (2004). Cell surface-associated elongation factor Tu mediates the attachment of *Lactobacillus johnsonii* NCC533 (La1) to human intestinal cells and mucins. Infect Immun.

[52] Bergonzelli GE, Granato D, Pridmore RD (2006). GroEL of *Lactobacillus johnsonii* La1 (NCC 533) is cell surface associated: potential role in interactions with the host and the gastric pathogen *Helicobacter pylori*. Infect Immun.

[53] Patel DK, Shah KR, Pappachan A (2016). Cloning, expression and characterization of a mucin-binding GAPDH from *Lactobacillus acidophilus*. Int J Biol Macromol.

[54] Katakura Y, Sano R, Hashimoto T (2010). Lactic acid bacteria display on the cell surface cytosolic proteins that recognize yeast mannan. Appl Microbiol Biot.

[55] Wang W, Jeffery CJ (2016). An analysis of surface proteomics results reveals novel candidates for intracellular/surface moonlighting proteins in bacteria. Mol Biosyst.

[56] Ehinger S, Schubert WD, Bergmann S (2004). Plasmin(ogen)-binding alpha-enolase from *Streptococcus pneumoniae*: crystal structure and evaluation of plasmin(ogen)-binding sites. J Mol Biol.

[57] Green ER, Mecsas J (2016). Bacterial secretion systems: an overview. Microbiol Spectrum.

[58] Ebner P, Rinker J, Götz F (2016). Excretion of cytoplasmic proteins in Staphylococcus is most likely not due to cell lysis. Curr Genet.

[59] Ebner P, Prax M, Nega M (2015). Excretion of cytoplasmic proteins (ECP) in *Staphylococcus aureus*. Mol Microbiol.

[60] Yang CK, Ewis HE, Zhang X (2011). Nonclassical protein secretion by *Bacillus subtilis* in the stationary phase is not due to cell lysis. J Bacteriol.

[61] Yang CK, Zhang XZ, Lu CD (2014). An internal hydrophobic helical domain of *Bacillus subtilis* enolase is essential but not sufficient as a non-cleavable signal for its secretion. Biochem Bioph Res Co.

[62] Boël G, Pichereau V, Mijakovic I (2004). Is 2-phosphoglycerate-dependent automodification of bacterial enolases implicated in their export?. J Mol Biol.

[63] Amblee V, Jeffery CJ (2015). Physical features of intracellular proteins that moonlight on the cell surface. PLoS One.

[64] Widjaja M, Harvey KL, Hagemann L (2017). Elongation factor Tu is a multifunctional and processed moonlighting protein. Sci Rep.

[65] Tacchi JL, Raymond BB, Haynes PA (2016). Post-translational processing targets functionally diverse proteins in Mycoplasma hyopneumoniae. Open Biol.

[66] Navarre WW, Schneewind O (1999). Surface proteins of Gram positive bacteria and mechanisms of their targeting to the cell wall envelope. Microbiol Mol Biol R.

[67] Schneewind O, Missiakas DM (2012). Protein secretion and surface display in Gram-positive bacteria. Philos T R Soc B.

[68] Scott JR, Barnett TC (2006). Surface proteins of Gram-positive bacteria and how they get there. Annu Rev Microbiol.

[69] Desvaux M, Dumas E, Chafsey I (2006). Protein cell surface display in Gram-positive bacteria: from single protein to macromolecular protein structure. FEMS Microbiol Lett.

[70] Antikainen J, Kuparinen V, Lähteenmäki K (2007). pH-dependent association of enolase and glyceraldehyde-3-phosphate dehydrogenase of *Lactobacillus crispatus* with the cell wall and lipoteichoic acids. J Bacteriol.

[71] Sánchez B, Bressollier P, Urdaci MC (2008). Exported proteins in probiotic bacteria: adhesion to intestinal surfaces, host immunomodulation and molecular cross-talking with the host. FEMS Immunol Med Mic.

[72] Daubenspeck JM, Liu R, Dybvig K (2016). Rhamnose links moonlighting proteins to membrane phospholipid in mycoplasmas. PLoS One.

[73] Antikainen J, Kuparinen V, Lähteenmäki K (2007). pH-dependent association of enolase and glyceraldehyde-3-phosphate dehydrogenase of *Lactobacillus crispatus* with the cell wall and lipoteichoic acids. J Bacteriol.

[74] (2013). Centers for Disease Control and Prevention, Antibiotic resistance threats in the United States. http://www.cdc.gov/drugresistance/threat-report-2013.

[75] Ventola CL (2015). The antibiotic resistance crisis: part 1: causes and threats. Pharm Ther.

[76] Matsuoka K, Kanai T (2015). The gut microbiota and inflammatory bowel disease. Semin Immunopathol.

[77] Dahlhamer JM, Zammitti EP, Ward BW (2016). Prevalence of inflammatory bowel disease among adults aged ≥18 years—United States, 2015. MMWR.

[78] Kinnby B, Booth NA, Svensater G (2008). Plasminogen binding by oral streptococci from dental plaque and inflammatory lesions. Microbiology.

[79] Heilmann C, Thumm G, Chhatwal GS (2003). Identification and characterization of a novel autolysin (Aae) with adhesive properties from *Staphylococcus epidermidis*. Microbiology.

[80] Sjostrom I, Grondahl H, Falk G (1997). Purification and characterization of a plasminogen-binding protein from *Haemophilus influenzae*. Sequence determination reveals identity with aspartase. BBA-Biomembranes.

[81] Candela M, Bergmann S, Vici M (2007). Binding of human plasminogen to Bifidobacterium. J Bacteriol.

[82] Beckmann C, Waggoner JD, Harris TO (2002). Identification of novel adhesins from Group B streptococci by use of phage display reveals that C5a peptidase mediates fibronectin binding. Infect Immun.

[83] Agarwal V, Kuchipudi A, Fulde M (2013). *Streptococcus pneumoniae* endopeptidase O (PepO) is a multifunctional plasminogen- and fibronectin-binding protein, facilitating evasion of innate immunity and invasion of host cells. J Biol Chem.

[84] Schreiner SA, Sokoli A, Felder KM (2012). The surface-localised α-enolase of *Mycoplasma suis* is an adhesion protein. Vet Microbiol.

[85] Matta SK, Agarwal S, Bhatnagar R (2010). Surface localized and extracellular Glyceraldehyde-3-phosphate dehydrogenase of *Bacillus anthracis* is a plasminogen binding protein. BBA-Proteins Proteom.

[86] Seifert KN, McArthur WP, Bleiweis AS (2003). Characterization of group B streptococcal glyceraldehyde-3-phosphate dehydrogenase: surface localization, enzymatic activity, and protein-protein interactions. Can J Microbiol.

[87] Bergmann S, Rohde M, Hammerschmidt S (2004). Glyceraldehyde-3-phosphate dehydrogenase of *Streptococcus pneumoniae* is a surface-displayed plasminogen-binding protein. Infect Immun.

[88] Winram SB, Lottenberg R (1996). The plasmin-binding protein Plr of group A streptococci is identified as glyceraldehyde-3-phosphate dehydrogenase. Microbiology.

[89] Jobin MC, Brassard J, Quessy S (2004). Acquisition of host plasmin activity by the Swine pathogen *Streptococcus suis* serotype 2. Infect Immun.

[90] Kainulainen V, Loimaranta V, Pekkala A (2012). Glutamine synthetase and glucose-6-phosphate isomerase are adhesive moonlighting proteins of *Lactobacillus crispatus* released by epithelial cathelicidin LL-37. J Bacteriol.

[91] Xolalpa W, Vallecillo AJ, Lara M (2007). Identification of novel bacterial plasminogen-binding proteins in the human pathogen *Mycobacterium tuberculosis*. Proteomics.

[92] Hickey TB, Thorson LM, Speert DP (2009). *Mycobacterium tuberculosis* Cpn60.2 and DnaK are located on the bacterial surface, where Cpn60.2 facilitates efficient bacterial association with macrophages. Infect Immun.

[93] Jarocki VM, Santos J, Tacchi JL (2015). MHJ_0461 is a multifunctional leucine aminopeptidase on the surface of *Mycoplasma hyopneumoniae*. Open Biol.

[94] Hussain M, Peters G, Chhatwal GS (1999). A lithium chloride-extracted, broad-spectrum-adhesive 42-kilodalton protein of *Staphylococcus epidermidis* is ornithine carbamoyltransferase. Infect Immun.

[95] Lechardeur D, Fernandez A, Robert B (2011). The 2-Cys peroxiredoxin alkyl hydroperoxide reductase c binds heme and participates in its intracellular availability in *Streptococcus agalactiae*. J Biol Chem.

[96] Boone TJ, Burnham CA, Tyrrell GJ (2011). Binding of group B streptococcal phosphoglycerate kinase to plasminogen and actin. Microb Pathogenesis.

[97] Fulde M, Bernardo-Garcia N, Rohde M (2014). Pneumococcal phosphoglycerate kinase interacts with plasminogen and its tissue activator. Thromb Haemostasis.

[98] Reddy VM, Suleman FG (2004). *Mycobacterium avium* superoxide dismutase binds to epithelial cell aldolase, glyceraldehyde-3-phosphate dehydrogenase and cyclophilin A. Microb Pathogenesis.

